# Cadmium accumulation, subcellular distribution and chemical fractionation in hydroponically grown *Sesuvium portulacastrum* [Aizoaceae]

**DOI:** 10.1371/journal.pone.0244085

**Published:** 2020-12-28

**Authors:** Mohammad Mazbah Uddin, Zhenfang Chen, Lingfeng Huang

**Affiliations:** Key Laboratory of the Ministry of Education for Coastal and Wetland Ecosystems, College of the Environment and Ecology, Xiamen University, Xiamen, China; Universidade de Santiago de Compostela, SPAIN

## Abstract

*Sesuvium portulacastrum* is a well-known halophyte with considerable Cd accumulation and tolerance under high Cd stress. This species is also considered as a good candidate of Cd phytoremediation in the polluted soils. However, the mechanism of Cd accumulation, distribution and fractionation in different body parts still remain unknown. Seedlings of *Sesuvium portulacastrum* were studied hydroponically under exposure to a range of Cd concentrations (50 μM or μmol/L to 600 μM or μmol/L) for 28 days to investigate the potential accumulation capability and tolerance mechanisms of this species. Cd accumulation in roots showed that the bio-concentration factor was > 10, suggesting a strong ability to absorb and accumulate Cd. Cd fractionation in the aboveground parts showed the following order of distribution: soluble fraction > cell wall > organelle > cell membrane. In roots, soluble fraction was mostly predominant than other fractions. Cd speciation in leaves and stems was mainly contained of sodium chloride and deionised water extracted forms, suggesting a strong binding ability with pectin and protein as well as with organic acids. In the roots, inorganic form of Cd was dominant than other forms of Cd. It could be suggested that sodium chloride, deionised water and inorganic contained form of Cd are mainly responsible for the adaption of this plant in the Cd stress environment and alleviating Cd toxicity.

## Introduction

In recent years, because of the rapid advancement of agricultural, electrical, chemical, and mining industries, a large amount of heavy metals has been released into the soil, water, and atmospheric environments, causing serious pollution to terrestrial and marine ecosystems [1, 2]. Owing to the low degradability and high stability of heavy metals in the environment, they can accumulate and be transported into food webs, resulting in a serious risk to human health [3, 4]. In coastal marine ecosystems, the remediation of heavy metals is of great concern for many countries and phytoremediation have become topics of great interest in recent years [5–7]. Phytoremediation is a plant based environmental remediation technology that proposed to remediate Cd from the polluted soils and water [8]. Phytoremediate plant species is the combination of higher Cd accumulation and tolerance with easy cultivation and fast growth [9].

Cadmium (Cd) is a non-essential metal and is very toxic to living organisms, even in very low concentrations [10]. Industrial sewage and agricultural activities are the main anthropogenic sources of Cd in the environment [11]. Cd has strong water solubility and mobility in the environment. Cd is easily absorbed by plants and when this Cd in plants accumulates, it can have a toxic effect on the plants including chlorosis and the inhibition of plant growth [12, 13]. Therefore, to remediate Cd from contaminated sites/waters, plants must maintain their growth in the stressed environment, accumulate metals in their roots, and lower mobility in the environment [14].

For remediation purposes, halophytes are more suitable than non-halophytes because they can maintain high growth and accumulation rates, and have a great tolerance of highly saline and toxic polluted environments [15]. Moreover, halophytes demonstrate a hyperaccumulator capacity to Cd accumulation due to the storage of metals in their cell walls and vacuoles [16, 17]. It can grow well at 100 mM to 400mM NaCl concentrations and it can further grow at 1000mM NaCl concentration without showing any symptoms in their leaves [18]. Several studies have suggested that higher salinity could reduce the toxic effects and lowering accumulation of Cd in *S*. *portulacastrum* and most of the studies conducted NaCl concentration was 200mM which was lower than the real field conditions [15–16, 18].

Recent studies have shown that *S*. *portulacastrum* has a great ability to accumulate Cd and is considered a phytoremediator species [16, 18]. Phytoremediation ability of plants depend on the subcellular distribution and chemical speciation of toxic metals in their tissues, which are important strategies for plants to reduce heavy metals toxicity. This partitioning occurs during the metals accumulation and provides vital information regarding detoxification and tolerance mechanisms [19]. It is well established that cell wall binding and vacuole compartmentation have the vital role in detoxification and tolerance mechanisms of hyperaccumulator plants under heavy metals stress [11, 20]. Several studies suggest that subcellular compartmentations vary in different species. For instance, Cd was compartmentalized in cell wall of root and mesophyll cell vacuole of *Thlaspi caerulescens* and *Arabidopsis halleri* [21, 22]; 40–90% of Cd was compartmentalized in roots of *Impatiens walleriana* as soluble fraction [23]; cell wall of roots and shoots was accumulated 54–62% of Cd in paddy rice [24]. Hydroponic experiment of barley genotypes suggest cell wall and vacuole compartmentation of Cd was vital for detoxification and tolerance [25].

Different forms of Cd (e.g. inorganic, water soluble, protein and pectin, phosphate and oxalate) extracted by different extractants and these extracted forms suggested the complex forms and mobility of Cd [11, 23, 26]. Previous studies of *S*. *portulacastrum* tolerance mechanisms under the Cd stresses were mostly in the narrow range of Cd concentrations such as 5μM to 25μM [18], 25μM to 50μM [16, 27] and 50 μM to 300 μM [28]. In our knowledge, there have no studies available on the tolerance and distribution mechanisms under the wide range (50 μM or μmol/L to 600μM or μmol/L) of Cd stress, which is very crucial for the remediation of Cd in the severe industrial, accidental pollution, mining and coastal polluted areas. Besides, a wide range of Cd stress indicates the significantly affected Cd concentration on *S*. *portulacastrum* and also suggests the feasibility and remediation ability of *S*. *portulacastrum* in the severe Cd polluted environments.

The objective of the present study was to investigate the speciation and distribution of Cd (50 μM or μmol/L to 600μM or μmol/L treatments) in tissues using the differential centrifugation and chemical reagent extraction method. In addition, the chemical binding morphology of Cd in roots, stems, and leaves was studied to elucidate the resistance mechanism of *S*. *portulacastrum* to Cd, as well as the distribution and chemical morphology of Cd in plant tissues. These results provide metals uptake, tolerance and fractionation ability and mechanisms of *S*. *portulacastrum* in the heavy metals polluted environment. Therefore, this study will increase our understanding of the application of *S*. *portulacastrum* in the coastal marine ecosystems for the remediation of heavy metals.

## Materials and methods

### Plant materials and treatment conditions

A sample of *S*. *portulacastrum* seedlings were collected from the Quanzhou seedling base station, Fujian Province. This plant has been cultivated and domesticated over the past 10 years at this base station. There was not required any written permission to collect the samples as this plants have higher growth, less valuable and distributed in the vast cultivated areas.

For the present study, 63 healthy plant was selected, from which a 3 cm long stem segment was taken with one node and two opposite leaves. The plant fragment was sterilised with calcium hypochlorite solution for 5 min and washed with distilled water. The plant fragment was reared under greenhouse conditions with Hoagland nutrient solution at a salinity of 20 ppt for 2 months. After that, 63 fragments of *S*. *portulacastrum* were selected for the experimental treatment from the reared plant. A similar growth and weight (10 g/fragment) condition of the plant fragments were maintained during the selection period. During the experimental treatment, plastic pots (17 cm × 14 cm) were used, which contained 2.5 L of Hoagland nutrient solution at a salinity of 20 ppt and pH of 6.5. To maintain the solution pH, 0.1 M sodium hydroxide or 0.1 M hydrochloric acid were added as pH regulators [29]. The plastic pots were divided into seven groups, with each group containing three pots. Cadmium chloride (CdCl_2_) was added at concentrations of 0 μM (control), 50 μM, 100 μM, 200 μM, 300 μM, 400 μM, and 600 μM. Three fragments of the selected plants were planted in each treatment pot and a floating bed was used to rear the plants. Each treatment contained three replicates. The growth conditions were a 16h light period at a light intensity of 250 μmol m^-2^S^-1^, 25±2°C temperature, and 60%–80% relative humidity. Nutrient solutions were changed every 4 days to maintain the correct Cd concentrations in solution.

### Quantification of Cd in plant tissues

After 28 days of treatment, three plants from each treatment were harvested and washed with deionised water. Subsequently, the height and weight of samples were measured, and the samples were divided into three parts: root, leaf, and stem. Root samples were soaked in cold Na_2_-ethylenediaminetetraacetic acid solution to remove all absorbed heavy metals from the root surface, and were then washed with Milli-Q water [16, 29]. The plant samples (root, leaf, and stem) were then oven dried at 105°C for 24h, after which the dried samples were ground with a mortar and pestle [16, 18, 29]. Following that, 0.1g of the samples were placed into a digestion tank and 3mL of concentrated HNO_3_ and 1mL of concentrated HClO_4_ was added [26]. The digestion tank containing the samples and chemicals was placed in an oven at 145°C for 24h. After complete digestion and cooling, the samples were transferred to 50mL centrifuge tubes and diluted up to 50mL with Milli-Q water.

### Cd in different plant cell fractions

In the plant tissues, Cd was separated into the following fractions: cell wall fraction, organelle fraction, membrane fraction, and soluble fraction, according to the differential centrifugation procedure [26–27, 30].

The extraction steps to obtain the different Cd fractions were as follows:

1. Cell wall fraction: A total of 0.5g freshly frozen plant samples were homogenised using precooled extraction buffer with 5% (w/v) containing 50mM Tris-HCl (pH 7.5), sucrose 250mM, and 1.0mM DL-dithiothreitol. After that, the homogenate was centrifuged at 3000 × g for 15min at 4°C and the precipitate constituted as cell wall fraction.
2. Organelle fraction: The filtrate solution was centrifuged at 15000 × g for 30 min and the pellet designated as organelle fraction.
3. Membrane fraction: The supernatant solution was further centrifuged at 100,000 × g for 30 minutes and the residue designated as membrane fraction.
4. Soluble fraction: The final supernatant solution.

All the steps were conducted at 4°C. All the residue fractions (cell wall fraction, organelle fraction, and membrane fraction) were dried and digested (mixture of HNO_3_ and HClO_4_, 3:1, v/v) with above mention method [26–27, 30].

### Sequential extraction of Cd speciation

The chemical speciation of Cd was conducted using the stepwise sequential extraction method [26] and different extraction media were considered as Cd fractions. The steps were as follows:

1. Addition of 80% ethanol, which mainly extracted inorganic chelated Cd of chloride, amino acid salts, nitrate, and nitrite fixing Cd substances.
2. Addition of distilled water, which extracted water-soluble organic acids and phosphate bound substances.
3. Addition of 1M NaCl to extract proteins and pectin binding Cd.
4. Addition of 2% acetic acid, which extracted insoluble Cd bound to phosphate compound.
5. Addition of 0.6M HCl, mainly extracted Cd oxalate.
6. Cd in the residual fraction.

Frozen plant tissues were homogenised in extraction solution with the help of mortar and a pestle, and the tissues were diluted at the ratio of 1:100 (w/v) [26]. Then, the homogenate was placed in a shaker at 25°C for 22h [26, 31]. The homogenate was then centrifuged at 5000 × g for 10 min, and the first supernatant was extracted [26]. After that, the residues were re-suspended twice in extraction solution and shaken again following the same procedure (5000 × g, 10 min) for 2h, and the supernatants were again extracted. All three supernatants were combined to obtain the first extraction. The precipitate was added to the next extraction media and the same methods followed to extract the remaining fractions. The extracts of each form were obtained using different extracting solutions, including ethanol extraction (F_E_), deionised water (F_W_), NaCl (F_NaCl_), acetic acid (F_HAc_), hydrochloric acid (F_HCl_), and residue (F_R_). The supernatant was poured into a 100 ml Erlenmeyer flask and heated at 70°C to obtain a constant mass and digested at 145°C with a mixture of HNO_3_ and HClO_4_ (3:1, v/v). The remaining residual tissues were digested with same above mention method.

### Quantification and quality control

The fractions and total concentration of Cd was quantified by Inductively Coupled Plasma Mass Spectrometer (ICP-MS, Agilent, 7500ce, USA). Instrumental and analytical accuracy was measured by analysing reagent bank, standard reference material and triplicate measurement. In this regard, we used standard reference material of Bush and Twing leaves (GBW07603). The average recovery rate from the standard reference material was 85% to 105%. The relative standard deviation of each sample was less than 5%. The detection limit of Cd was 0.009ppb.

### Data analysis

In order to evaluate the phytoremediation capacity of *S*. *portulacastrum*, the bio-concentration factor (BCF) and translocation factor (TF) of plant parts were calculated, using the following formulas:

BCF=heavymetalsconcentrationinsamples/heavymetalsconcentrationinsolution

TF=heavymetalsconcentrationinabovegroundtissues/heavymetalsconcentrationintheroot.

One-way analysis of variance was conducted to compare the treatments and Tukey’s test was used to detect the significant differences among treatments. Statistical analyses were conducted using SPSS 19.0 (SPSS for Windows, SPSS Inc.).

## Results and discussion

### Effect of Cd on the biomass of *S*. *portulacastrum*

**Fig 1:**
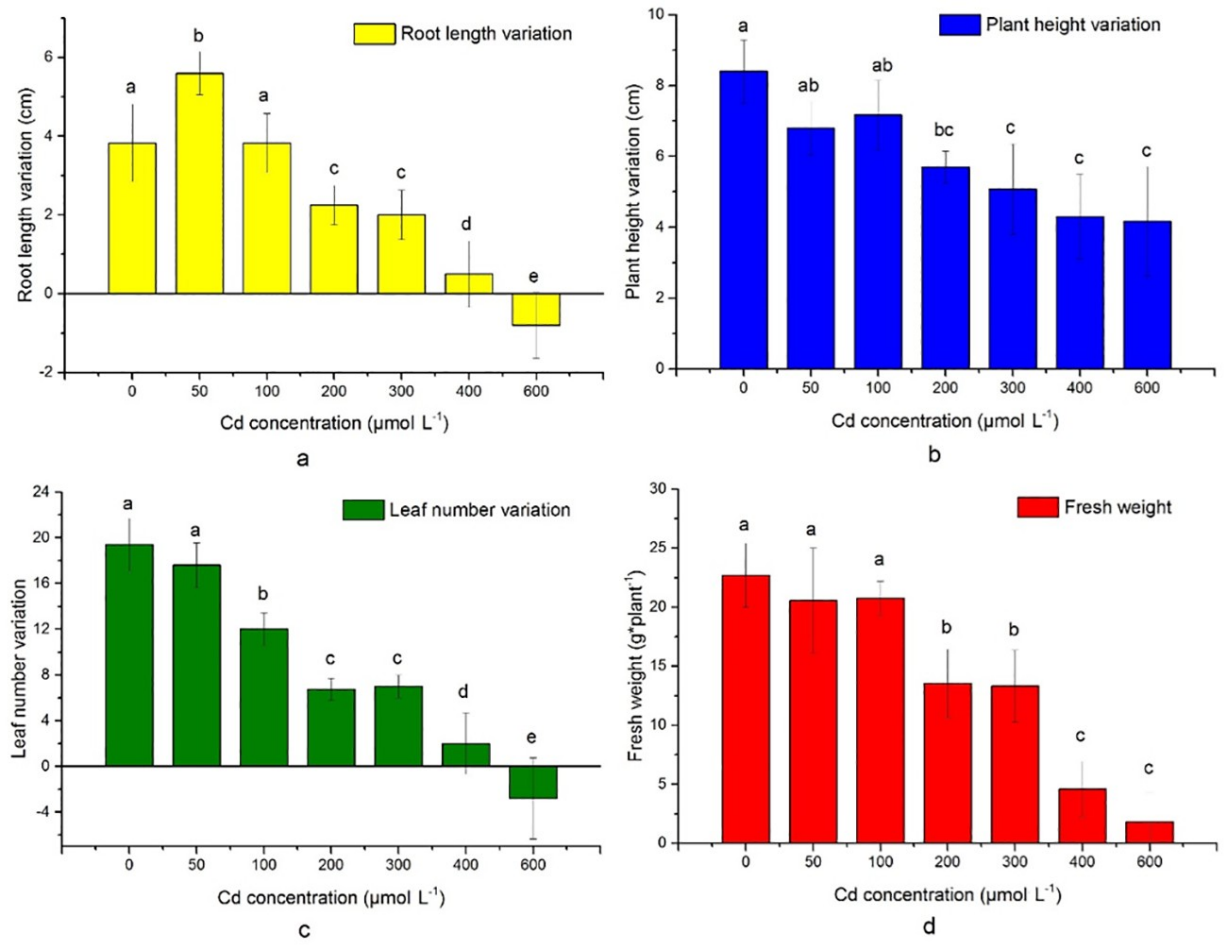
Effects on different Cd treatments on physical parameters of *S*. *portulacastrum* (Different letters in different treatments suggest significant difference (P<0.05) between treatments according to ANOVA and Turkey test).

Cd treatments significantly affected the root length, plant height, leaf number and fresh weight (total biomass) of *S*. *portulacastrum* (Fig 1a–1d). There was a significant decrease (P < 0.05) in root length with increasing Cd concentrations; however, the control plant (0 μM) had a shorter root length than that of the 50 μM Cd treatment had (Fig 1a), indicating that low Cd concentration (50 μM) did not retard the growth of plants. Similar result was reported by Feng et al. (2018) [18] in a hydroponic solution experiment, where higher biomass and growth was found under the Cd treatments of 5 μM and 10 μM concentration. In addition, low concentration of Cd might increase the Zn^2+^ transport competition from nutrients solution [32, 33]. Zn is the important micronutrient for plant growth. Several studies suggest that Zn and Cd showed competition in plant nutrients uptake and increasing biomass due to dilution effect [33, 34]. Plants higher biomass and growth depends on nutrient uptake, minimise oxidative stress, reduce the damage of cellular metabolism [35]. Under the low Cd stress *Sesuvium* can alter redox condition and energetic status for support higher growth and biomass [36]. In the greater Cd stress, this plant cannot take essential nutrients from the soil or solution [36].

In the 200 μM, 300 μM, 400 μM, and 600 μM Cd treatments, the plant height decreased by 32%, 40%, 48%, and 50%, respectively (P < 0.05) more than that of the control (Fig 1b), demonstrating that higher Cd concentrations retarded the growth and height of *S*. *portulacastrum*. A significant difference in total biomass (P < 0.05) was observed between the 50 μM and 100 μM treatment with the 200 μM, 300 μM, 400 μM, and 600 μM treatments (Fig 1d). Total plant biomass decreased by 92% and 80% (P < 0.05) in the 400 μM and 600 μM Cd concentration treatments, respectively (Fig 1d).

Under Cd stress, halophytes are capable of changing the anatomical mechanisms of their leaves, water use efficiency, physiological and biochemical pathways [35, 36]. Halophytes have the ability to minimize the Cd stress through the mechanism of exclusion, excretion and accumulation [36]. In addition, halophytes show considerable Cd tolerance owing to their detoxification mechanisms such as the formation of phytochelatins, metallothioneins, and enzymatic and non-enzymatic antioxidant [37]. Besides this plants can produce osmoprotectants that can maintain water potential gradient and prevent toxic ions to damage cell structures [37].

For example, in a previous study, in comparison with non-halophytes plants, such as *Brassica juncea* that showed toxicity symptoms (e.g. defoliation and yellowish colour of leaves) under 100 μM Cd concentration, halophytes such as *Cakile maritima* did not show any symptoms under this concentration [38]. In another study, *Zea mays* showed lower biomass of leaves and chlorophyll pigment under Cd stress of 25 μM concentration [39].

Plant total biomass did not change significantly under the stress of the 50 μM and 100 μM Cd treatments (Fig 1). However, under the 200 μM Cd treatment, plant growth was inhibited significantly (Fig 1). Ghnaya et al. (2005) [28] and Taamalli et al. (2014) [38] reported similar findings, where growth of *S*. *portulacastrum* and *C*. *maritima* were not affected under 200 μM Cd stress. Under 200 μM Cd stress treatment, S. *portulacastrum* can maintain its chlorophyll content, proline levels, and photosystem II mechanism more efficiently than other plants [28]. However, in higher concentration treatments, the Cd stress affects the redox reaction of the plants, exacerbates the reactive oxygen free radicals, and destroys the cell structure, which leads to the retardation of growth [27, 40]. However, the adaptability of *S*. *portulacastrum* under 200 μM Cd stress indicates that it might be an ideal candidate for the phytoremediation of Cd from areas with pollution levels are less than 200 μM.

### Bioaccumulation and translocation of Cd by *S*. *portulacastrum*

**Fig 2:**
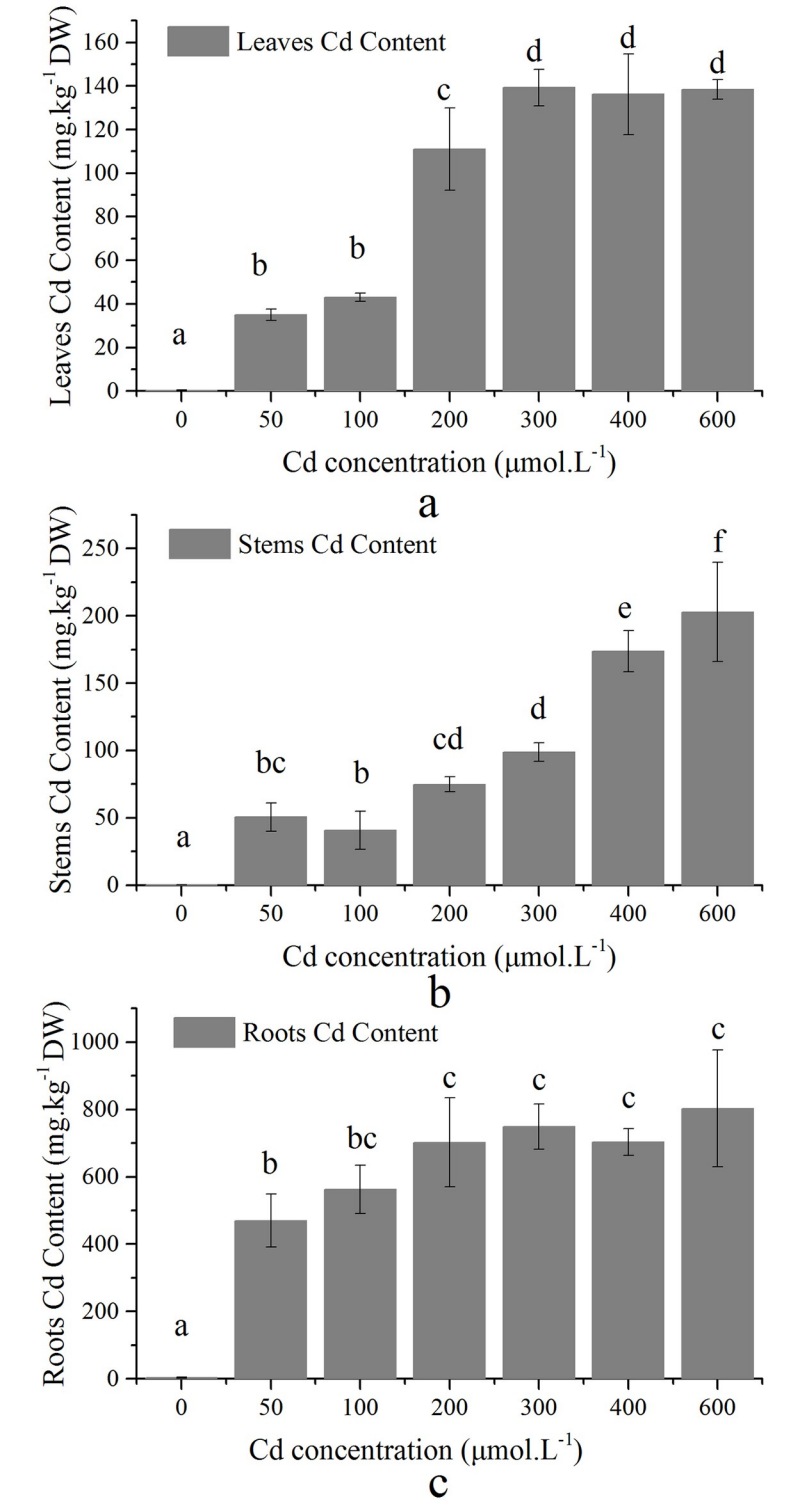
Variations of Cd concentrations in *S*. *portulacastrum* (a: Leaves; b: Stems; c: Roots).

Cd content in leaves, stems and roots was significantly (P < 0.05) increased with increasing Cd concentration treatments (Fig 2a–2c). The accumulation of Cd in leaves showed a significant (P < 0.05) difference among the 100 μM, 200 μM, and 300 μM Cd treatments, with the highest accumulation observed in the 300 μM Cd treatment (Fig 2a). In stems, the accumulation increased gradually with the increasing Cd concentrations (Fig 2b). The Cd content in the roots was higher than that in the stems and leaves (Fig 2c). However, among the 200 μM to 600 μM Cd concentrations, accumulation in the roots did not change significantly (P > 0.05), indicating that the higher Cd treatment reduced accumulation in the plant, which is related to the excluding capacity of this species as well as a higher tolerance to adverse conditions [9]. In both roots and aboveground tissues, the BCF values were higher than 1, and the BCF of the roots was higher than that of the aboveground tissues (Table 1), indicating that this species has a strong ability to accumulate Cd in its tissues (leaves and stems), and thus has great potential for Cd remediation [18].

**Table 1:** Bio-concentration factor (BCF) and translocation factor (TF) of Cd in *S*. *portulacastrum*.

	Cd concentration (μM)					
	50	100	200	300	400	600
TF (%)	9.11±1.10	7.45±0.55	13.25±1.05	15.89±1.02	22.02±1.87	21.24±1.44
Root BCF	83.89±14.1a	50.24±6.43bc	31.35±5.88bc	22.28±2.00d	15.70±0.88d	12.95±2.57d
Above ground tissues BCF	7.64±0.92a	3.74±0.28bc	4.15±0.33bc	3.54±0.23bc	3.46±0.29bc	2.54±0.17d

(Different letters in the same row suggest significant difference (P<0.05) between treatments according to ANOVA and Turkey test)

In addition, BCF of roots and above ground tissues were significantly (P<0.05) decreased with increasing Cd stress (Table 1). The higher accumulation capacity of roots suggests the use of this species as a potential phytoremediator species for Cd-polluted environments [35]. It also shows that from a concentration of 200 μM to 600 μM Cd, accumulation in the roots increased; however, this accumulation was not significant, indicating that under higher stress conditions, this species adopted an exclusion strategy instead of accumulation to prevent cell damage [9, 28, 38].

Higher Cd concentration in stems than that in leaves was most probably related to the maintenance of photosynthetic capacity and increasing other essential nutrients resulting in the reduction of Cd in leaves [13, 18]. Several studies have shown that higher salinity increased the accumulation and translocation of heavy metals in the roots and tissues of *S*. *portulacastrum* [16, 27, 29]. Thus, S. *portulacastrum* is a highly suitable Cd phytoremediator for sea water or saline coastal areas and lagoons.

The BCF and TF of heavy metals are important criteria for evaluating the plant potentiality for the removal of metals from the contaminated environment, and a value > 1 indicates the accumulation ability of a plant, which differentiates plant species into different categories such as accumulator, hyperaccumulator, and so on [8, 31, 41]. A plant is considered a hyperaccumulator if the shoot concentration of heavy metals is higher than 1000 mg/kg, including a BCF above 1000 and a TF greater than 1 [42, 43]. A hydroponic experiment (solution) of Cd stress on *Limna minor* suggests that BCF was >1000 during 1.5 to 2.5 mg/L of Cd exposed for 72h [44]. Therefore, *Arabidopsis halleri* and *Thlaspi caerulescens* also showed hyperaccumulator ability during hydroponic Cd stress study (solution) as their shoots accumulation >1000 mg/kg of Cd [21, 45]. *Arabidopsis halleri*, *Thlaspi caerulescens*, *Thlaspi praecox*, *Sedum alfredii* and *Solanum nigrum* are recognized as Cd hyperaccumuator species [46].

In the present study, both root and shoot concentrations were lower than 1000 mg/kg and root concentrations were close to the hyperaccumulator range. Therefore, *S*. *portulacastrum* is not a hyperaccumulator, even though its BCF values were higher than 10 (Table 1), which suggests its potential as a strong accumulator and phytostabiliser of Cd in contaminated areas [13, 41, 47]. The accumulation in roots was higher than that in aboveground tissues and their TF was lower than 1. This may be due to the self-protective mechanism of this species to accumulate more in its root vacuoles than in its aboveground tissues [13, 48]. The common strategy of metal-tolerant plants is to accumulate higher amounts of toxic metals in their roots than in their aboveground tissues. A similar result was observed in *Bechmeria nivea*, where higher accumulation of Cd was reported in the roots than in the aboveground tissues [49]. This strategy is a tolerance mechanism to Cd of the studied plant. Therefore, BCF of roots was lower and TF was greater than present study, suggesting that roots are the main accumulator of Cd from solution condition [20]. Overall, the accumulation of Cd in tissues under the hydroponic soil condition was lower than the solution hydroponic experiment of *S*. *portulacastrum* [50]. This result possibly indicates the potential of phytoremediator of Cd from the coastal polluted waters.

However, in the present study, the continual growth and bioaccumulation ability under Cd stress indicated the phytoextraction and phytostabilisation capacity of *S*. *portulacastrum* in polluted environments.

### Subcellular distribution of Cd in *S*. *portulacastrum*

**Fig 3:**
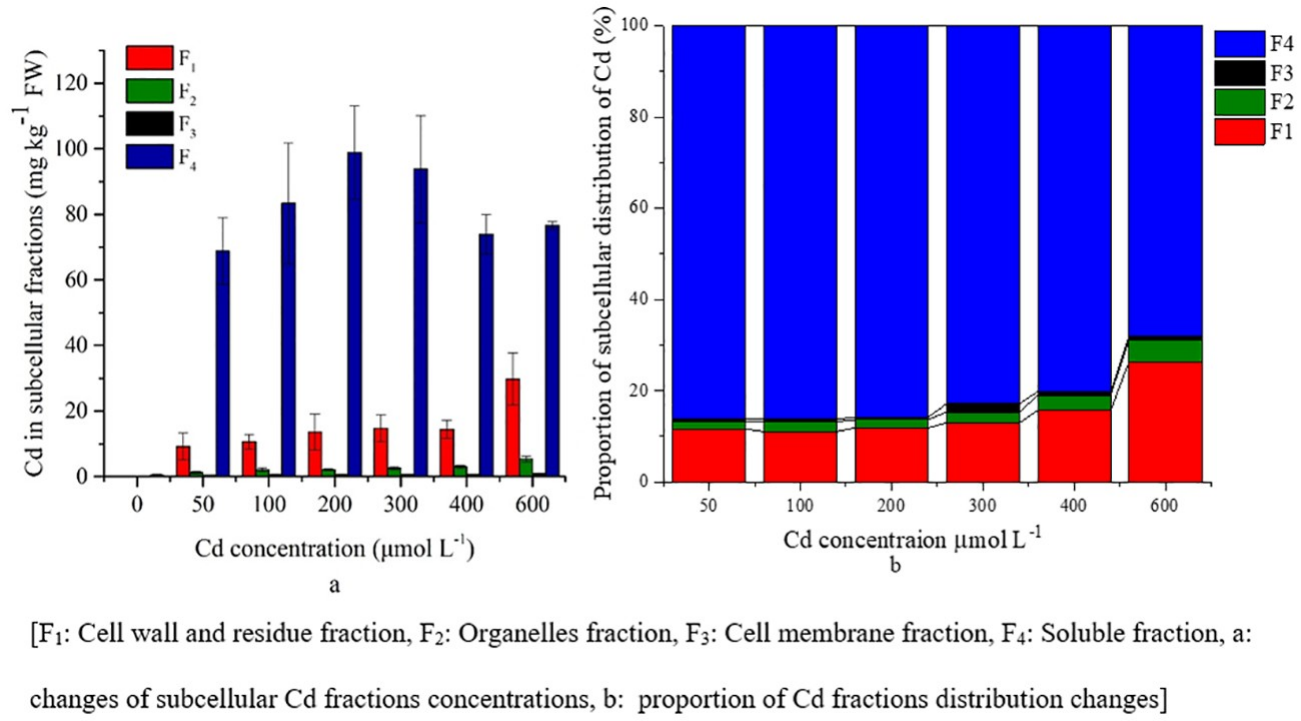
Subcellular distribution of Cd in *S*. *portulacastrum* roots.

The subcellular distribution of Cd concentration in the roots, stems and leaves was different because of the different Cd treatments. In roots, more than 80% of the Cd fraction in the cellular distribution was the soluble fraction and the cell wall fraction was approximately 12% (Fig 3a and 3b).

There was a significant (P<0.05) increase of cell wall and residue and organelle fraction concentrations with increasing Cd treatments (S1 Table). The plant root showed an increased soluble fraction until the 200 μM Cd treatment, and then gradually decreased (from 86% to 68%), whereas the cell fraction increased (from 10%–12% to 14%) under higher Cd treatments (Fig 3a and 3b).

**Fig 4:**
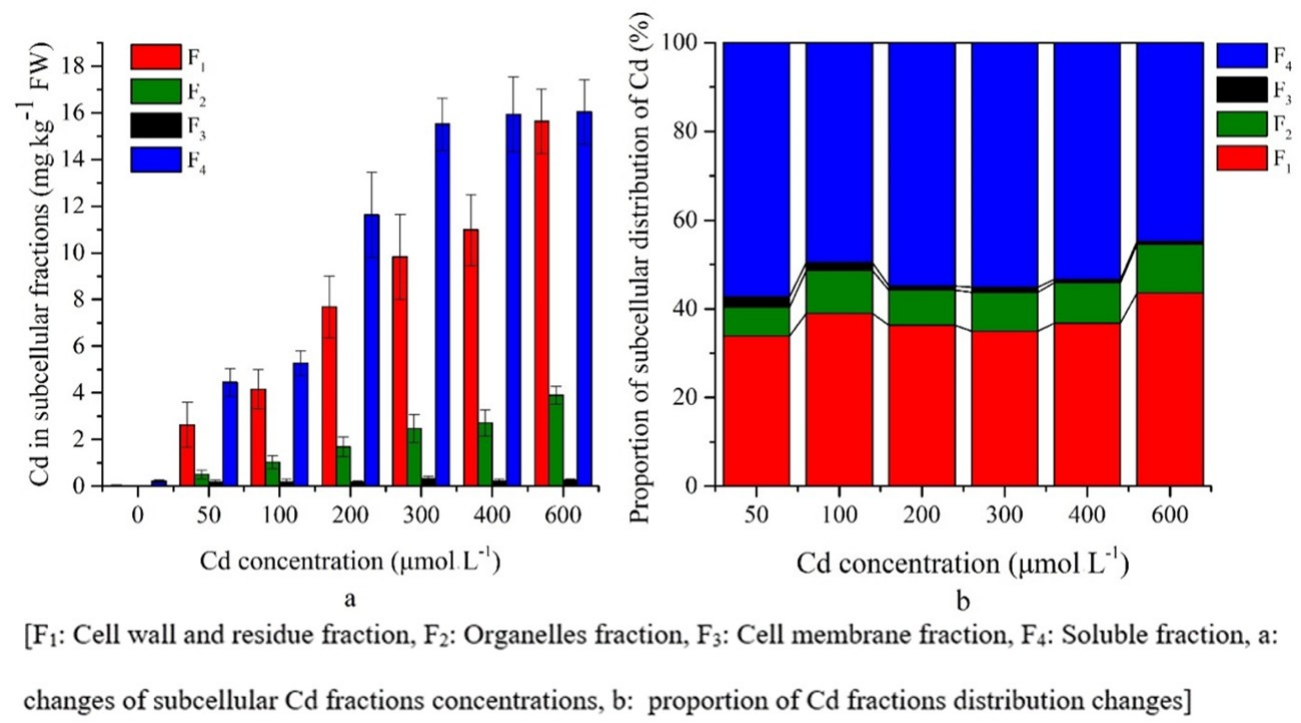
Subcellular distribution of Cd in *S*. *portulacastrum* stems.

In stems, the Cd distribution fraction was similar to that in leaves, where approximately 90% of Cd was distributed as the cell wall and residual fractions, as well as the soluble fraction. There was a significant (P<0.05) increase of cell wall and residue, organelle and soluble fractions concentrations with increasing Cd treatments (S1 Table). However, the cell wall fraction was 10% higher and the soluble fraction was 12% lower with increasing Cd stress than that of the leaf fraction (Fig 4a and 4b).

**Fig 5:**
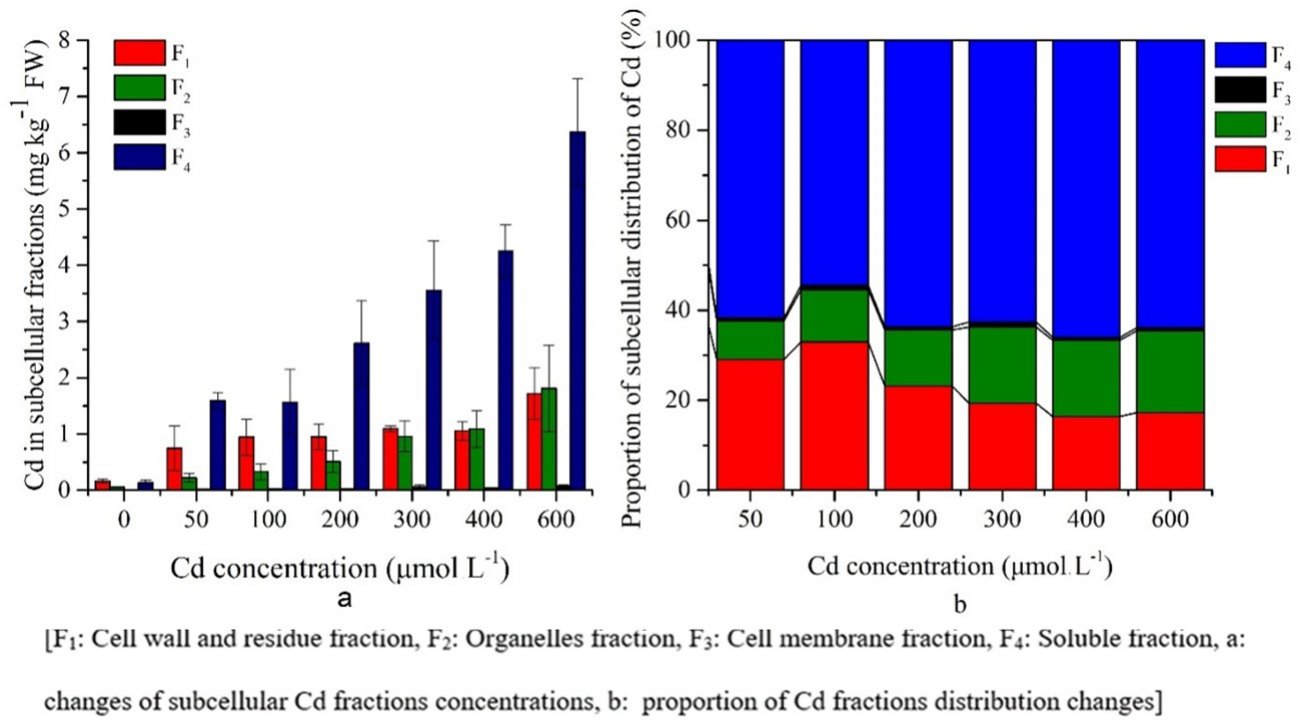
Subcellular distribution of Cd in *S*. *portulacastrum* leaves.

The Cd soluble fraction had a higher concentration in leaves and gradually increased significantly (P<0.05) with Cd treatments (Fig 5a, S1 Table); therefore, under adverse conditions, the plants changed their distribution of metals form, which was more tolerable than the other fractions.

In addition, the cell wall, residue, and organelle fractions were increased significantly (P<0.05) in the higher Cd treatments; however, the change of concentration was lower than that of the soluble fraction (Fig 5a, S1 Table). In the cells of the leaves, approximately 90% of the Cd distribution fraction was in the cell wall and residual fraction as well as in the soluble fraction (Fig 5b). There was no concentration change for the cell membrane fraction with increasing Cd stress except 600 μM Cd treatment (Fig 5a and 5b, S1 Table).

Several studies have shown that the subcellular distribution of heavy metals plays an important role for the tolerance of heavy metals and the detoxification mechanism of plants [16, 18, 29, 47]. In the present study, the cell wall and soluble fractions contributed 16.41%-44.30% and 37.96%-66.07%, 9.37%-43.63% and 44.73%-85.75%, 11.03%–26.42% and 67.96%–86.42% respectively (Figs 3–5). The increasing Cd concentrations showed a slight increase in the cell wall fraction; however, the soluble fraction decreased (Figs 3–5). Therefore, Cd was bound to the cell walls as CdCl_2_ complexes and there was decreased free Cd ions resulting in less accumulation in the symplasm [16, 27]. The cell wall is composed of poly sugars and proteins, and contains carboxyl, hydroxyl, and aldehyde, with Cd having a strong affinity to bind with the apoplasts of these pectins, and thus reduce the toxicity at symplastic sites [16, 29]. Moreover, soluble fractions increased until the 200 μM Cd treatment, which indicted that this concentration might trigger plants to begin binding Cd fractions to the cell wall and organelles of plants to increase tolerance. Similar results have been observed in different plant species, both in halophytes, e.g. *Artiplex halimus* and *Tamarix smyrnensis*, and non-halophytes, e.g. *Bechmeria nivea* and *Porphyra yezoensis* [30, 43, 51].

However, in the non-halophyte species, the cell wall fraction was dominant over the soluble fraction [30, 43]. In the present study, the soluble fraction (67.96%–86.42%) was dominant in *S*. *portulacastrum*, indicating that the soluble form of Cd was mostly stored in the vacuoles, which is a fundamental strategy of this plant for Cd stress tolerance [9, 16, 27]. In addition, this strategy decreased the available soluble form of Cd that could bind with other plant organelles [30]. The transportation and storage of soluble Cd in vacuoles, organic acids, and sulfur are essential during this strategy [30]. Therefore, the Cd in the aboveground parts was lower than that in the roots in all treatments, with the leaves having the lowest concentration, indicating the Cd binding affinity of chloroplast in leaves [30, 52]. Lai (2015) [23] and Xin and Huang (2014) [53] showed that more than 76% of Cd was in soluble form in the roots of *Impatiens walleriana* and chilli (hot pepper), which was similar to the results from our present study. In contrast, in comparison with soil hydroponic experiment of Cd stress on *Siegesbeckia orientalis* showed lower accumulation of soluble fraction in leaves, stems and roots than present study [20].

The distribution of the Cd fractions in the stems and leaves showed that the cell wall fractions were higher than those in than roots and their concentrations increased with increasing Cd stress; however, the soluble fractions were lower than those in the roots (Figs 3–5). These findings show that the cell wall is a prominent barrier to stress conditions. Moreover, the vacuole of stems is an effective Cd storage compartment under stress conditions.

### Chemical fractionation of Cd in *S*. *portulacastrum*

**Fig 6:**
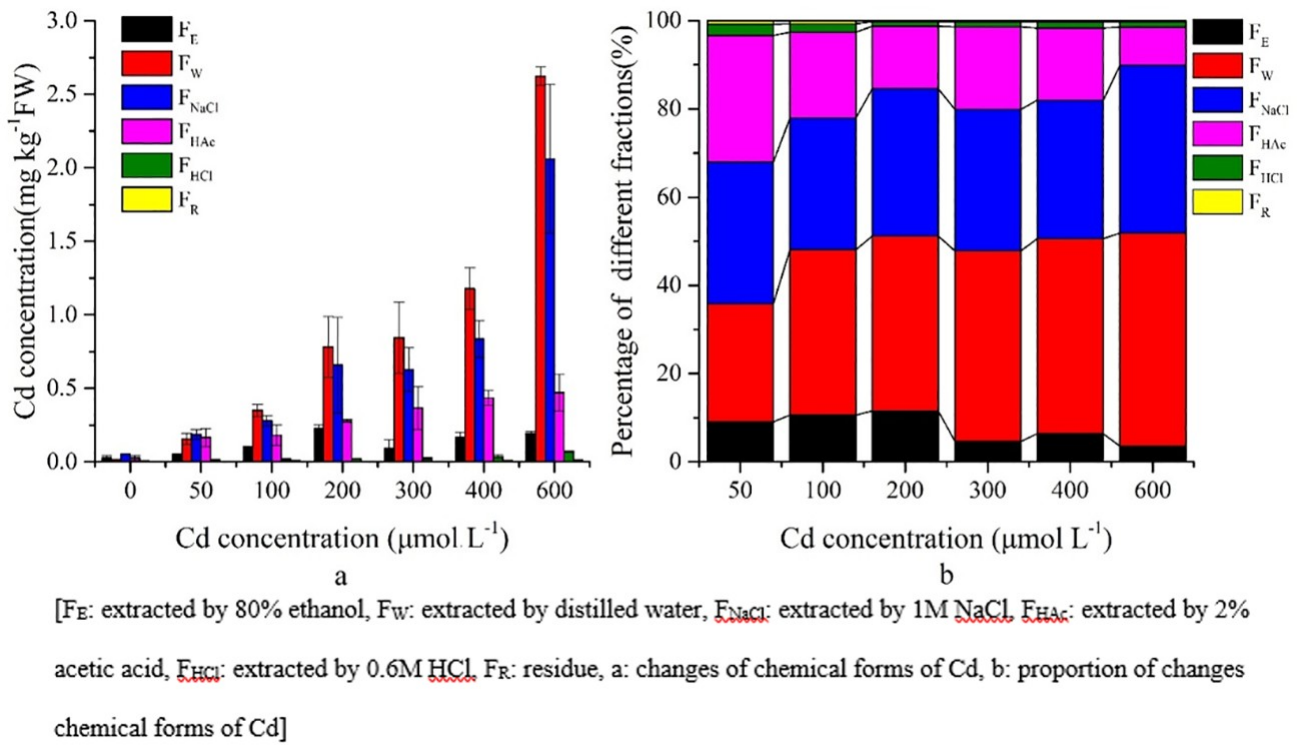
Chemical forms of Cd and proportions in *S*. *portulacastrum* leaves.

**Fig 7:**
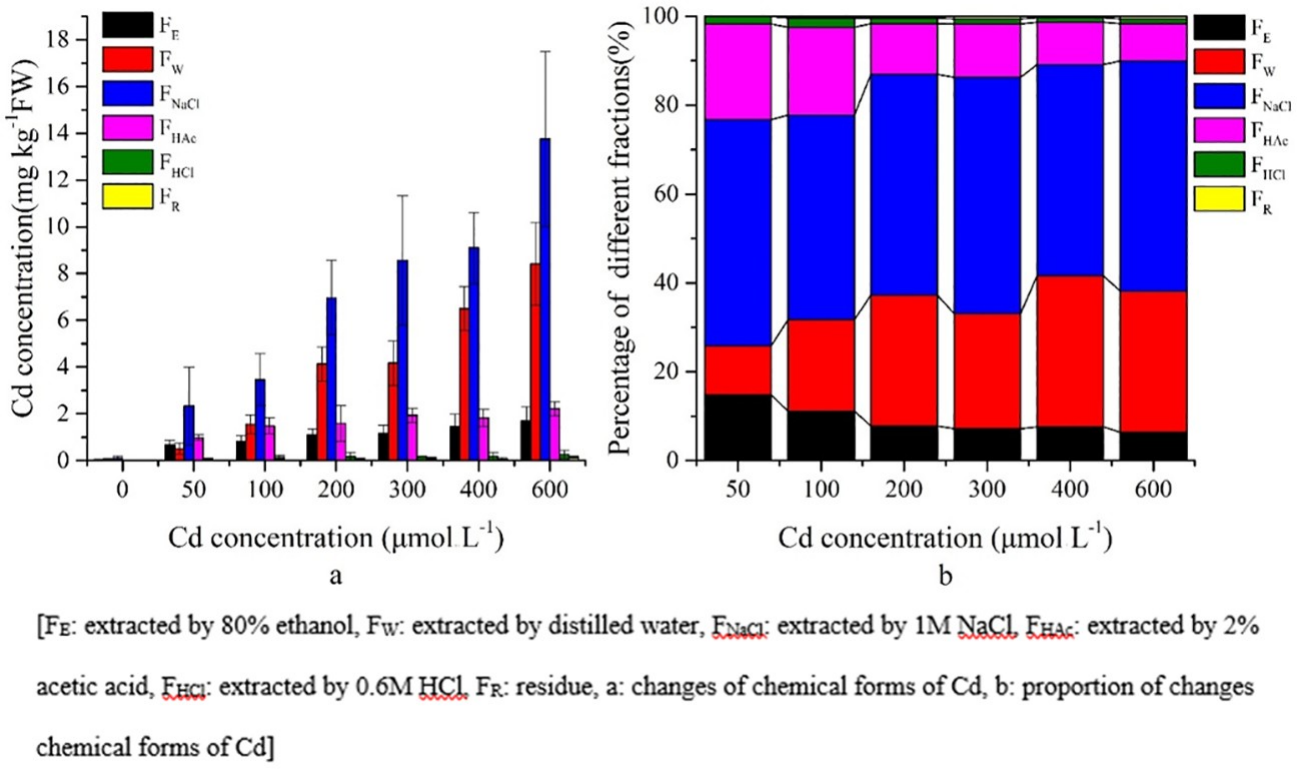
Chemical forms of Cd and proportions in *S*. *portulacastrum* stems.

**Fig 8:**
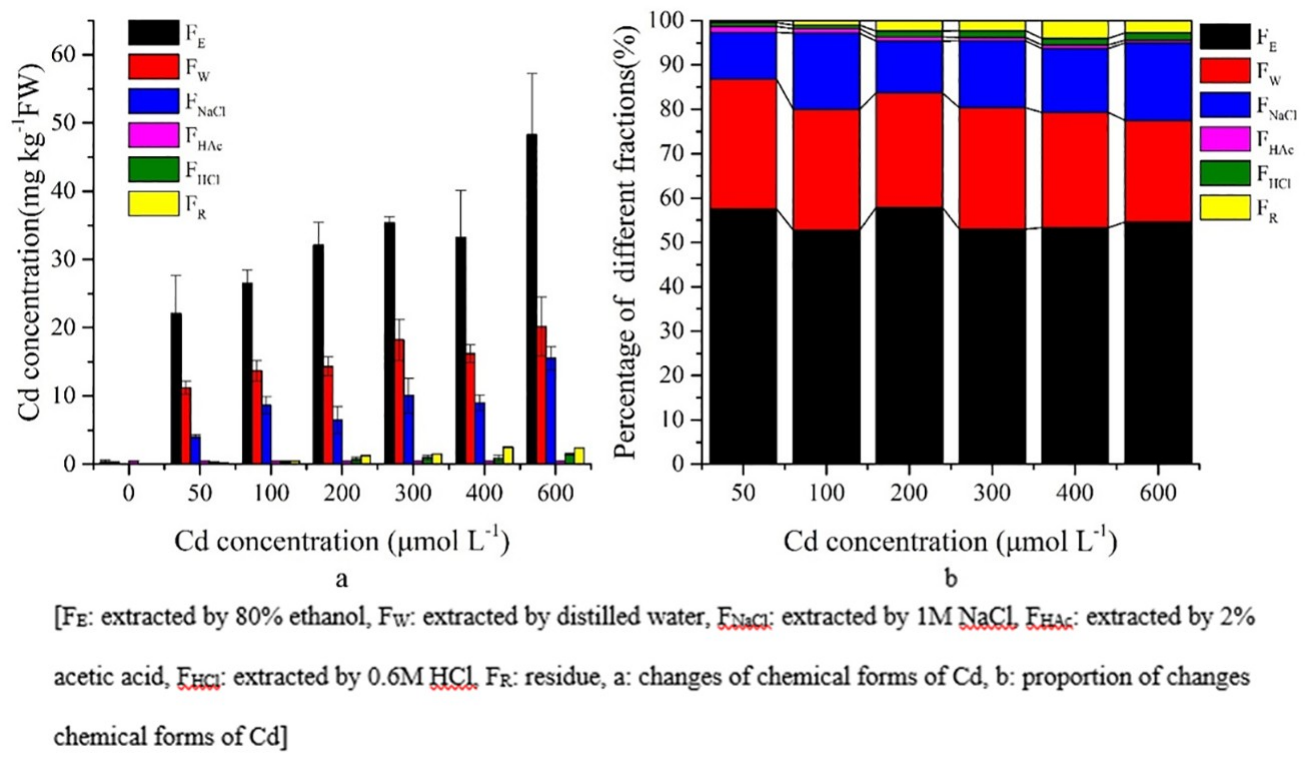
Chemical forms of Cd and proportions in *S*. *portulacastrum* roots.

In general, F_W_, F_NaCl_ and F_HAc_ forms of Cd were dominant in leaves, stems, and roots of *S*. *portulacastrum* (Figs 6–8). The concentration of the extracted Cd forms in *S*. *portulacastrum* decreased as follows: roots > stems > leaves (Figs 6–8). In the leaves, approximately 86.80% of Cd forms were F_W_, F_NaCl_, and F_HAc_ (Fig 6a and 6b).

The proportion of Cd forms of F_W_ and F_NaCl_ increased significantly (P<0.05) with increasing Cd concentration (Fig 6a and 6b, S2 Table). However, F_HAc_ and F_E_ decreased significantly (P<0.05) from 28.69% to 8.66% and from 9% to 4%, respectively, with increasing Cd treatments (Fig 6a and 6b, S2 Table).

Chemical forms of Cd in stems decreased as follows: F_NaCl_ > F_W_ > F_HAc_ > F_E_ > F_HCl_ > F_R_ and their concentration increased in the higher Cd treatments (Fig 7a). However, the proportion of different fractions showed that F_NaCl_ increased significantly (P<0.05) with increasing concentration treatments and contained the highest proportion between 46% and 53% (Fig 7b), whereas the F_W_ proportion increased significantly (P<0.05) by 18.36% with increasing concentration. The F_E_ and F_HAc_ proportions decreased from 15% to 6% and from 20% to 8%, respectively (Fig 7b).

The chemical forms of Cd in the root tissues were very distinct from the composition of leaves and stems. The F_E_ extracted form of Cd had a higher concentration in root tissues than in the stems and leaves, and their proportion did not change with the increasing concentration treatments; however, their concentrations did increase significantly (P<0.05) at higher Cd concentration treatments (Fig 8a and 8b, S2 Table).

In addition, F_W_ and F_NaCl_ extracted forms of Cd did not change their proportion in the higher Cd concentrations but significantly (P<0.05) increased their concentration (Fig 8a and 8b, S2 Table). In general, in the roots, three main forms F_W_, F_NaCl_, and F_E_ of Cd accounted for approximately 95% of the total chemical forms (Fig 8b).

In plants, the toxicity of heavy metals, biological functions, and Cd migration are related to the chemical forms of Cd, which are extracted by different chemical solutions [30]. In terms of migration and toxicity, inorganic and organic forms (F_E_ and F_W_ respectively) of Cd have higher mobility, resulting in higher toxicity for plants than that of other forms of Cd (F_NaCl_ and F_HAc_) [10]. In the present study, the highest percentage of Cd in the leaves and stems were in F_W_, F_NaCl_, and F_HAc_ (Figs 6 and 7). These fractions might be responsible for the adaptation of *S*. *portulacastrum* under a Cd stress environment. Moreover, in roots, the higher percentage of the Cd inorganic form (F_E_) was dominant over the other forms of Cd (Fig 8). Therefore, the higher percentage of the F_E_ form of Cd and the much lower percentage of F_HAc_ and F_Nacl_ are responsible for this plant being able to adapt to the Cd stress environment. Furthermore, during increased Cd stress, roots adopted more inorganic forms of Cd (F_E_) and translocated water-soluble organic phosphate intergrade form (F_W_), and pectin and protein binding form (F_NaCl_) to the leaves and stems to adapt to the adverse Cd stress conditions. This zstrategy shows that compartmentation in vacuoles and translocation to cell walls are the crucial detoxification mechanisms of this plant under a Cd stress environment [16, 27, 30]. In addition, comparison with soil hydroponic Cd treatments on *Impatiens walleriana* and *Siegesbeckia orientalis* showed that F_NaCl_ was dominant forms of Cd in root tissues, whereas F_E_ was dominant form of Cd in present study [20, 23]. This result indicates that chemical forms of Cd in plants tissues not only depends on physiology but also in the contaminated environments (e.g. soil, soil type, solution) [20, 23, 50].

The present study not only provided information regarding the distribution and speciation of Cd in tissues of *S*. *portulacastrum*, but also identified the phytoextraction capacity of the studied species. In this context, we are aware that this hydroponic experiment results cannot completely show what occurs in field experiments. However, to obtain reliable and accurate information of the tolerance by a plant to heavy metals and distribution patterns, this hydroponic experiment provides improved baseline information [10]. The studied plant is non-edible, has high biomass, and quick growth; therefore, it is a suggested species for potential Cd remediation. In addition, because of its strong phytoextraction capacity, this plant should be completely harvested from the polluted environment after the remediation of the experimental sites.

## Conclusions

*S*. *portulacastrum* reflected a high tolerance to Cd-polluted environment. Plant biomass had no significant effect when Cd concentrations reached 200 μM but from 300 μM to 600 μM growth significantly decreased. The tolerance to the higher Cd treatments, obtained from sequential extraction, showed that detoxification mechanisms such as free Cd ions binding with pectin and protein, formation of organic acids, and compartmentation and storage in vacuoles, plays a prominent role in adverse polluted environment. In addition, the BCF (> 10) and TF (< 1) indicated that S. portulacastrum is not a hyperaccumulator but is potentially considered a phytoextractor and phytostabiliser of heavy metals in coastal polluted environments.

## Supporting information

S1 Table Subcellular distribution of mean Cd fractions (mgKg^-1^ FW) in leaves, stems and roots of *S*. *portulacastrum* under different Cd treatments. (PDF)

S2 Table Average chemical forms of Cd fractions (mgKg^-1^ FW) in leaves, stems and roots of *S*. *portulacastrum* under different Cd treatments. (PDF)
