# Supplementary material for: Cadmium accumulation, subcellular distribution and chemical fractionation in hydroponically grown Sesuvium portulacastrum [Aizoaceae]
Source: PLoS One. 2020 Dec 28;15(12):e0244085. doi: 10.1371/journal.pone.0244085 (PMC7769616; doi:10.1371/journal.pone.0244085)
Supplement: S1 Table — (PDF) [file pone.0244085.s001.pdf]

		Cd subcellular fractions (mgkg^{-1} FW)			
Organs	Treatments (µML^{-1})	F1	F2	F3	F4
Leaves	Cd 50	0.75a	0.22a	0.02ab	1.59a
	Cd100	0.94a	0.33a	0.03a	1.56a
	Cd200	0.95b	0.51a	0.03a	2.61ac
	Cd 300	1.10b	0.96ab	0.07ab	3.55c
	Cd 400	1.06b	1.09ab	0.04ab	4.25c
	Cd 600	1.72c	1.81b	0.07b	6.36d
Stems	Cd 50	2.63a	0.51a	0.18a	4.45a
	Cd100	4.16a	1.03ac	0.19a	5.49a
	Cd200	7.69b	1.68ce	0.19a	11.62b
	Cd 300	9.83b	2.47e	0.32a	15.51c
	Cd 400	10.98b	2.72e	0.24a	15.93c
	Cd 600	15.63c	3.90f	0.26a	16.03c
Roots	Cd 50	9.27a	1.24a	0.41a	68.84a
	Cd100	10.66a	2.12ab	0.52a	83.38a
	Cd 200	13.66a	2.13ab	0.57a	98.90a
	Cd 300	14.77a	2.61ab	2.08a	93.82a
	Cd 400	14.43a	3.08b	0.72a	73.90a
	Cd 600	29.77b	5.40c	0.94a	76.60a

(Different letters in same organ means significant differences ($P<0.05$) among different treatments according to ANOVA and Turkeys test; FW denoted fresh weight)
