# Supplementary material for: Cadmium accumulation, subcellular distribution and chemical fractionation in hydroponically grown Sesuvium portulacastrum [Aizoaceae]
Source: PLoS One. 2020 Dec 28;15(12):e0244085. doi: 10.1371/journal.pone.0244085 (PMC7769616; doi:10.1371/journal.pone.0244085)
Supplement: S2 Table — (PDF) [file pone.0244085.s002.pdf]

		Chemical forms of Cd mgKg^{-1} FW					
Organs	Treatments (μML^{-1})	F_E	F_W	F_{NaCl}	F_{Hac}	F_{HCl}	F_R
Leaves	Cd 50	0.05a	0.15a	0.19a	0.17a	0.02a	0.00
	Cd100	0.10a	0.35a	0.28a	0.18a	0.02a	0.01a
	Cd200	0.23b	0.78b	0.66a	0.28ab	0.02ab	0.00
	Cd 300	0.07a	0.84a	0.63a	0.37ab	0.02ab	0.00
	Cd 400	0.17c	1.18c	0.84a	0.43b	0.04bc	0.01a
	Cd 600	0.19d	2.62b	2.06b	0.47b	0.07d	0.01a
Stems	Cd 50	0.68a	0.51a	2.33a	0.99a	0.08	0.00
	Cd100	0.84ab	1.55a	3.46ab	1.49a	0.15a	0.03a
	Cd200	1.10ab	4.13b	6.96abc	1.59a	0.18a	0.05a
	Cd 300	1.16ab	4.17bc	8.65bd	1.94a	0.18a	0.10ab
	Cd 400	1.46ab	6.51c	9.10cd	1.82a	0.19a	0.06a
	Cd 600	1.69b	8.42d	13.75d	2.22b	0.28a	0.16b
Roots	Cd 50	22.09a	11.20a	4.06a	1.78a	0.33a	0.17a
	Cd100	26.54a	13.72ab	8.66ab	3.26ac	0.39a	0.51b
	Cd 200	32.11a	14.32 ab	6.51ab	4.53acd	0.75ab	1.28c
	Cd 300	35.37ab	18.25 ab	10.07b	6.08bcd	1.01ab	1.51e
	Cd 400	33.29ab	16.20 ab	9.01ab	5.27bcd	0.91ab	2.50f
	Cd 600	48.25b	20.16b	15.51c	7.96d	1.51b	2.39g

(Different letters in same organ means significant differences ($P<0.05$) among different treatments according to ANOVA and Turkeys test; FW denoted fresh weight)
